# Giant hamartomatous polyp of the uterine cervix with heterologous mesenchymal tissue in a child: a case report 

**DOI:** 10.1186/s13256-021-02878-3

**Published:** 2021-06-01

**Authors:** Esmatullah Esmat, Haider Ali Malakzai, Mujtaba Haidari, Ahmed Maseh Haidary, Merwaise Baha, Jamshid Abdul-Ghafar

**Affiliations:** 1 Department of Pathology and Clinical Laboratory, French Medical Institute for Mothers and Children (FMIC), Kabul, Afghanistan; 2 Department of Internal Medicine, Medical Collage, Balkh University, Balkh, Afghanistan

**Keywords:** Cervix, Polyp, Hamartoma, Cartilage, Female genital tract

## Abstract

**Background:**

Polyps of the uterine cervix are one of the most common benign hyperplastic lesions occurring in the female genital tract that usually arise from the endocervical canal and are believed to be the result of reactive changes due to long-standing chronic inflammation, multiparty, and foreign bodies. Cervical polyps are usually small in size (less than 4 cm) that are commonly found in adult women; however, a few cases of giant polyps and the rare occurrence of these lesions in children have also been reported. Heterotopias and malignant transformation in cervical polyps are considered to be very rare.

**Case presentation:**

We present the case of a 10-year-old Afghan girl with a giant pedunculated mass protruding out of the uterine cervix that was accompanied by abdominal pain and mass sensation. The mass was completely excised by surgical intervention and the specimen was submitted for histopathological evaluation. Upon gross and microscopic examination, the characteristic findings of a hamartomatous polyp with heterologous mesenchymal tissue in the form of mature cartilage were seen. To the best of our knowledge, this is the first case of a giant (17.5 cm) hamartomatous polyp of the uterine cervix in this age group.

**Conclusion:**

Giant hamartomatous cervical polyps rarely occur in patients below 10 years of age. The majority of these lesions are benign; however, a few cases with malignant transformation are also reported, which demands elaborate investigations into the etiopathogenesis and nature of the lesions.

## Background

Polyps of the uterine cervix (UC) are one of the most common benign lesions occurring in the female genital tract that usually arise from the endocervical canal and are believed to be the result of reactive changes due to long-standing chronic inflammation, multiparty, and foreign bodies [1]. A cervical polyp (CP) is considered to be a focal overgrowth of UC usually lined by combined endocervical glandular and ectocervical squamous epithelium with characteristic loose, edematous stroma having thick-walled blood vessels [2]. Reactive hyperplastic changes of the epithelium along with stromal chronic active inflammation composed of eosinophils, neutrophils, and plasma cells are a common microscopic finding in UP compared to normal endocervical tissue which harbors fewer inflammatory cells [2]. Although the main factors that stimulate hyperplasia and the chronic inflammatory process in the pathogenesis of UP remain unclear, abnormal hormonal stimulation and infection are proposed to be the responsible agents, respectively [2]. Heterotopias are rarely reported in UC in the form of sebaceous tissue, neuroglial tissue, cervical melanosis, heterologous cartilage, and a case of hamartomatous endocervical polyp containing mature adipose tissue with islands of cartilage [3]. CPs are more commonly found in women over the age of 20 and are less likely to occur before menarche, which is probably due to the abnormal local response to increased levels of estrogen or local congestion of cervical blood vessels [4]. Although nearly all CP are benign, incidence of malignancy is reported in approximately 0.1% of cases [1]. On the other hand, giant CP greater than 4 cm in size are extremely rare with only a few cases described in the literature including a case of enormous (17 cm) CP in a 26-year-old nulliparous woman followed by a case of large (12 cm) CP in a multiparous 48-year-old woman complicated by massive vaginal bleeding [5]. Here we present a unique case of gigantic CP with heterogeneous mesenchymal tissue in a 10-year-old girl.

## Case presentation

A 10-year-old Afghan girl presented to the hospital with lower abdominal pain and mass sensation. Radiological investigation revealed a huge mass attached to the UC with well-defined borders. Upon surgical intervention, the entire pedunculated mass protruding out of cervical canal was removed and submitted for histopathological evaluation.

**Fig. 1:**
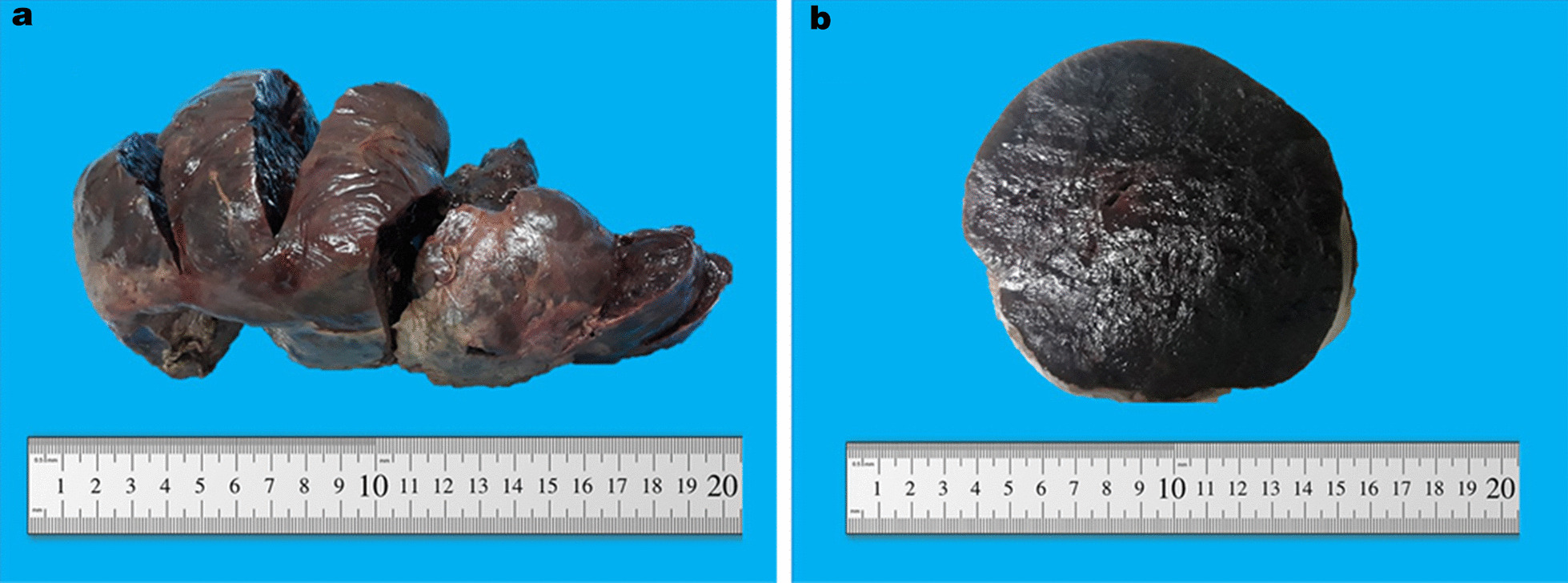
Gross appearance of the polyp showing a well-circumscribed dark-brown smooth external surface (**a**) with a dark-brown homogenous shiny cut surface (**b**)

**Fig. 2:**
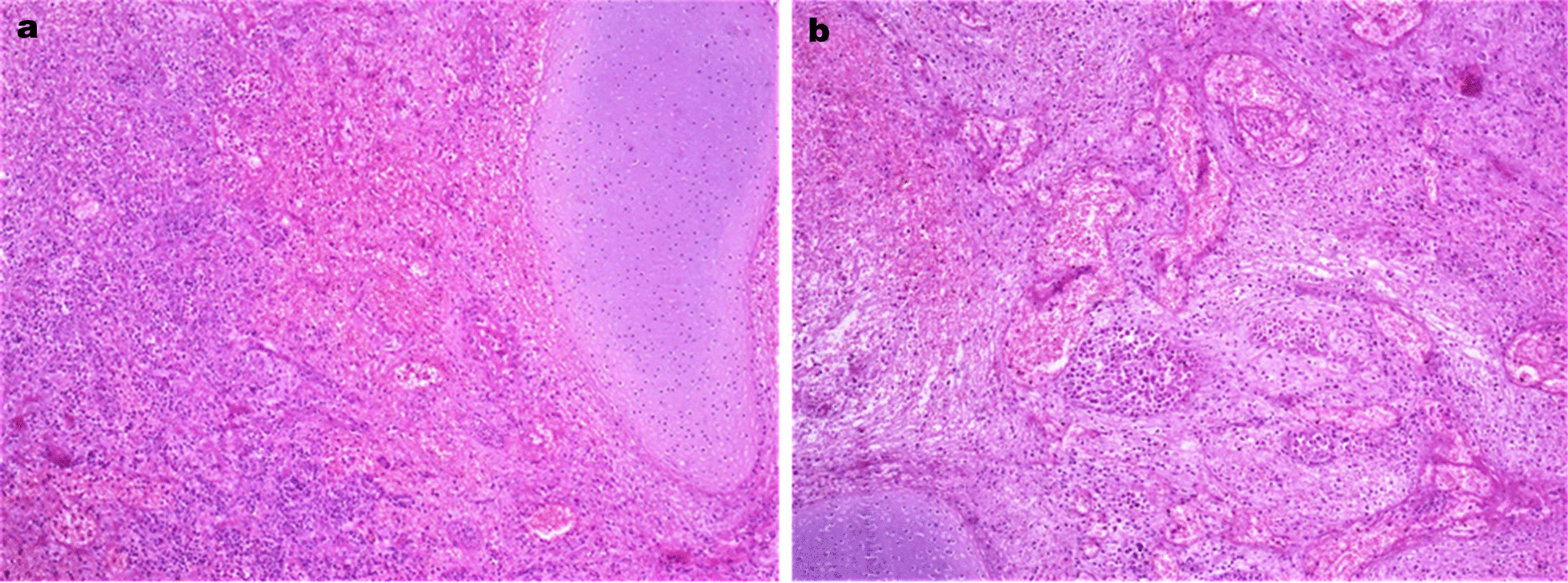
Intermediate power microscopic presentation of the polyp exhibiting mature cartilage island surrounded by loose, edematous, hemorrhagic stroma (**a**) with moderate chronic inflammation and multiple dilated blood vessels filled with red blood cells (**b**)

The gross examination of the specimen revealed a well-circumscribed dark-brown firm mass with the gigantic size of 17.5 cm in diameter that showed a smooth external surface (Fig. 1a). Upon serial sectioning, it exhibited a dark-brown homogenous shiny cut surface (Fig. 1b). Microscopic evaluation of the mass showed endocervical tissue composed of mature cartilage islands surrounded by loose, edematous, and hemorrhagic stroma (Fig. 2a) with moderate chronic inflammatory cell infiltrate mainly comprising lymphocytes and numerous dilated blood vessels filled with red blood cells (Fig. 2b). No evidence of atypia or mitotic activity was seen in the sections examined and on the basis of these findings the diagnosis of a hamartomatous polyp was made.

## Discussion

CPs are relatively common benign lesions of the UC harboring focal hyperplasia of the endocervical columnar epithelium which often present as asymptomatic hyperplastic masses and are usually found in parous women aged 30–50 years [1, 6]. In spite of the fact that the majority of CPs are asymptomatic, some of them present with postcoital bleeding, metrorrhagia, postmenopausal bleeding, menorrhagia, and leukorrhea [4]. Giant CPs refer to polyps greater than 4.0 cm in size with only a few cases reported in the literature; sometimes these CPs clinically mimic malignancy [7, 8]. Despite the unknown etiology, several factors are proposed to be involved in the pathogenesis of CP including multiparty, foreign bodies, and long-standing chronic inflammation [2, 6]. In our case, there was dense infiltration of chronic inflammatory cells mainly composed of lymphocytes with extensive hemorrhage, probably due to torsion.

Malignant transformation in CP is very rare with the prevalence of malignancy reported in 0.1% [4, 5], dysplasia in 0.5%, and reactive atypia in 1.6% of cases [4]. Inflammatory changes are documented in 27.7% of polyps, metaplasia in 13.6%, and microglandular hyperplasia in 6.8% [4]. In the current case, although no sign of atypia, dysplasia, or malignancy was seen, islands of mature cartilage along with thick-walled vascular structures in the stroma were largely present. The age of the patient in our case is a unique factor that separates it from the previously described ones with the largest ever reported CP measuring 17.5 cm in diameter. Although CPs frequently develop in the adult and adolescence periods, incidence of giant polyps in patients below the age of 10 years is extremely uncommon with only one case of a 5-year-old girl reported, who presented with a large (5 cm) endocervical polyp [5, 9, 10]. Despite the usual occurrence of CPs, only a few cases are reported with heterologous elements, such as mature cartilaginous and adipose tissues [3]. The treatment of choice for smaller CPs is dilatation and curettage; however, for larger polyps surgical excision is recommended [4]. CPs rarely recur and have a recurrence rate of 6.2% in the reported cases [4].

## Conclusions

We present a rare case of 10-year-old girl with a giant hamartomatous CP harboring heterologous mesenchymal tissue such as mature cartilage. Although a few cases of large (greater than 4 cm) CP are reported, almost all of the them were found in adults that adds to the uniqueness of our case accompanied by the gigantic size of the polyp. The majority of CPs are benign; however, malignant transformation is also reported, which demands further investigations into the etiopathogenesis and nature of the lesions.

## Data Availability

All the generated data are included in this article.

## Abbreviations

UC: Uterine cervix
CP: Cervical polyp
